# Mesocorticolimbic Dopamine‐Reward Tract Alterations and the ‘Dark Side’ of Methamphetamine Addiction

**DOI:** 10.1111/adb.70186

**Published:** 2026-09-16

**Authors:** Robert J. Roy, Ken T. Wakabayashi, Robert J. R. Blair, Nicholas A. Hubbard

**Affiliations:** ^1^ Department of Psychology University of Nebraska–Lincoln Lincoln Nebraska USA; ^2^ Center for Brain, Biology, and Behavior Lincoln Nebraska USA; ^3^ Department of Psychiatry Virginia Commonwealth University Richmond Virginia USA

**Keywords:** dopamine, mesocorticolimbic, methamphetamine, reward

## Abstract

Mesocorticolimbic circuits are central to theories of methamphetamine and other addictions. White‐matter fibres are a necessary component of those circuits. However, the extent to which mesocorticolimbic white‐matter tracts are altered in methamphetamine addiction is unknown. The current study addressed this gap as well as sought to determine relationships with neurofunctional and behavioural deficits concerning nondrug rewards. Multimodal MRI was acquired from 92 people with problematic methamphetamine‐use experience (MA^+^) and 98 methamphetamine‐naïve controls (MA^−^). Diffusion tractography mapped probabilistic fibre paths connecting midbrain presynaptic dopamine hubs to striatal and medial orbitofrontal areas highly sensitive to rewards. MA^+^ exhibited microstructural alterations (via fractional anisotropy [FA]) along this mesocorticolimbic tract—in particular, white‐matter segments running through anterior limb of the internal capsule (ALIC_FA_: MA^+^ > MA^−^) and near the medial orbitofrontal target area (mOFC_FA_: MA^+^ < MA^−^). In addition, task‐fMRI revealed that MA^+^ exhibited reduced activations to monetary rewards within the striatal and mOFC area versus MA^−^. Moreover, MA^+^ participants reported they attributed lower hedonic value and lesser motivational salience to broader nondrug rewards/pleasant experiences relative to MA^−^. Further, within MA^+^ participants, those exhibiting decreased ALIC_FA_ or mOFC_FA_ reported attributing lower hedonic value to nondrug rewards/experiences relative to their peers—with a similar correlation found for ALIC_FA_ and motivational‐salience reports. Our findings identified microstructural alterations in a mesocorticolimbic white‐matter tract plausibly supporting reward‐related dopaminergic signalling in people experienced with methamphetamine. Such alterations may play a role in the lower valuation of nondrug rewards and diminished motivation to pursue typical/nondrug activities associated with methamphetamine use.

## Graphical abstract

Nondrug rewards are commonly discounted, and the motivation to pursue healthful, drug‐free activities is often diminished in addiction. This study found that people with problematic methamphetamine‐use experience exhibited microstructural alterations in bundles of a mesocorticolimbic white‐matter tract implicated in reward processing. Microstructural coherence within the altered bundles was associated with their attributions of hedonic value to nondrug rewards and their tendencies to ascribe motivational salience to drug‐free activities.

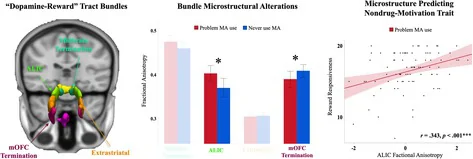

Mesocorticolimbic circuits are often invoked in neurobiological theories of methamphetamine (MA) and other addictions [1, 2, 3, 4]. Yet, while white‐matter fibres are a necessary component of those circuits, the precise nature and extent of MA‐related alterations within mesocorticolimbic white matter are unclear. Moreover, it remains unknown whether such alterations underpin phenomena of the so‐called ‘dark side’ of MA addiction [2, 4]. Namely, the apparent grey‐matter hyposensitivity to nondrug rewards along with behavioural deficits concerning the valuation and motivational salience of those rewards associated with MA use [5, 6, 7]. Closing these gaps has clear relevance for addiction theory and may have clinical significance, as alterations in both the structures and functions of related circuitry are associated with addiction treatment outcomes [8, 9].

Alterations in mesocorticolimbic circuits are posited to contribute to the development and maintenance of addiction [1, 2, 3, 4]. These circuits summarily include projections from midbrain dopamine hubs to striatal and medial/orbital prefrontal regions, as well as those regions' projections to midbrain. Functional, morphological and neurochemical alterations within mesocorticolimbic grey matter have been catalogued in murine models of MA addiction [7, 10, 11, 12]. Some homologous grey‐matter alterations have also been observed in people with MA use disorder [1]. Yet little is known regarding potential alterations within their mesocorticolimbic white matter.

A principal white‐matter tract that could underlie mesocorticolimbic circuits is comprised of anterior fibres of the medial forebrain bundle. In humans, these fibres have been observed to run from dopamine‐rich midbrain regions, projecting through the anterior limb of the internal capsule (ALIC) near striatal nuclei, like nucleus accumbens, and extend into orbital/medial prefrontal white‐matter fields [13]. The limited diffusion MRI work available suggests alterations in proximal fibres in MA use disorder [9, 14]. Those works demonstrated decreased microstructural coherence (via fractional anisotropy [FA]) in white‐matter tracts connecting midbrain to nucleus accumbens within mixed samples of people with stimulant use disorders (including MA) compared to controls. Inasmuch as FA indexes the white‐matter microstructures supporting neurotransmission (e.g., axon packing density, myelin content, etc.), these findings suggest a relative dysconnectivity that may extend more widely across mesocorticolimbic circuits.

Microstructural alterations within white‐matter tracts can undermine the typical functions of the grey‐matter regions these fibres connect [15]. However, it is not known whether mesocorticolimbic microstructural alterations relate to grey‐matter dysfunction observed among people with MA use disorder. This dysfunction includes a hyposensitivity of their mesocorticolimbic grey‐matter regions to nondrug rewards [5, 16, 17]. For instance, one task‐fMRI study evidenced reduced striatal activations during the receipt of pleasant tactile stimulation (i.e., soft touch) for people with MA use disorder compared to controls [5]. Another found that people with MA use disorder exhibited reduced orbitofrontal activations during an incentivized decision‐making task (i.e., ‘correct’ or ‘incorrect’ feedback) [16]. Notably, hyposensitivity to nondrug rewards may hold translational significance, as reduced striatal activations to such stimuli (e.g., monetary gains) have been associated with relapse following treatment for MA use disorder [8].

Mesocorticolimbic microstructural alterations may also bear relevance to the deficits in the valuation or motivational salience of nondrug rewards found alongside chronic MA and other stimulant experience [6, 7, 18, 19]. Such microstructure–behaviour relationships have not yet been documented among people with MA use disorder. However, links between mesocorticolimbic white matter and similar behaviours have been observed in other contexts. For instance, acute stimulation of ventral striatal or ventral midbrain white‐matter fields produces rapid behavioural changes characteristic of mesocorticolimbic activation in people with depression, such as invigorating approach behaviours and appetitive motivation [20, 21]. Diffusion‐MRI findings have also identified relationships between mesocorticolimbic white‐matter tracts and reward valuation. Indeed, people with a lower volume of white‐matter connections between ventral tegmentum and orbitofrontal cortices report anticipating less pleasurable outcomes (i.e., lower hedonic valuation) from rewarding or otherwise enjoyable experiences compared to those with greater volumes [22]. More generally, mesocorticolimbic white‐matter connections support signal transmission among its constituent grey‐matter regions, which has been observed to regulate reward sensitivity and reward pursuit in preclinical investigations [23, 24, 25].

The evidence available points towards mesocorticolimbic circuit alterations associated with MA use. However, evidence of alterations within the white‐matter fibres that may ultimately underpin mesocorticolimbic circuits remains undiscovered. We sought to address this gap by identifying a mesocorticolimbic white‐matter tract plausibly supporting reward‐related signalling between midbrain dopamine hubs, striatum and orbitofrontal cortex. We leveraged open metanalyses of both microarray gene expressions and functional neuroimaging to delineate midbrain grey‐matter enriched with presynaptic dopamine markers along with striatal and medial orbitofrontal (hereafter, corticolimbic) areas confirmed to be active across more than 900 studies of reward stimuli or processes [26]. These volumes of interest were used in probabilistic tractography to recover a mesocorticolimbic ‘dopamine‐reward’ tract across participants with problematic MA‐use experience (MA^+^) and MA‐naïve controls (MA^−^).

We tested whether MA^+^ exhibited microstructural alterations (via FA) along this tract compared to MA^−^. Like prior works [9, 14], FA was examined along this tract's trajectory in lieu of global tract averages because diffusion indices reliably fluctuate along many tracts and, as such, may afford a more complete understanding of microstructural differences [27]. Task fMRI was used to test whether MA^+^ exhibited the hypothesized hyposensitivity to nondrug (i.e., monetary) rewards in corticolimbic grey‐matter volumes compared to MA^−^. We also tested the extent to which altered white‐matter microstructure in the recovered tract was related to MA^+^ participants' grey‐matter responses to nondrug rewards as well as their attributions of hedonic value and motivational salience to nondrug rewards/experiences.

## 1 Method

### 1.1 Participants and Procedure

Study procedures were approved by the University of Nebraska‐Lincoln Institutional Review Board, informed consent was obtained before study procedures, and participants were compensated for their time. Data were collected as part of a large‐scale, longitudinal project examining relationships between brain function and MA use. Only relevant study procedures are described here. Study recruitment, inclusion/exclusion and procedures have been described previously [28, 29].

Participants were English‐speaking adults, ages 19–60 years. Prospective cases were identified from a larger cohort of people who use illicit drugs recruited via community sampling [30]. Pre‐screening assessed: MA use within 6 months, preferred substance, MRI‐contraindications and willingness to abstain from alcohol/illicit substances for at least 12 h before study sessions. Trained staff then interviewed pre‐screened cases regarding their recent MA use and DSM‐5 criteria adapted for MA use disorder. The MA^+^ group comprised people reporting symptoms that met DSM‐5 clinical threshold for MA use disorder within the past 12 months and/or reporting using MA at least 20 of the past 30 days via the Risk Behaviour Survey [31]. Community sampling and the inclusive MA^+^ group definition sought to sample a wide range of problematic MA‐use behaviours and associated traits. MA^−^ participants were identified via advertisements and a local registry and reported neither an MA‐use history nor a substance use disorder history.

Exclusion criteria were current pregnancy, chronic or uncontrolled illness, current psychosis, uncorrected visual impairments, neurological conditions, neurodevelopmental disorders (except ADHD), trauma resulting in extended unconsciousness and MR contraindications (e.g., ferrous implants). Neither psychiatric conditions nor polysubstance use were exclusionary as these are common among the broader population of people who use MA and exclusion risks limiting study generalizability [32, 33]. Abstinence from alcohol and/or illicit substances at least 12 h prior to participation was required. This was confirmed at intake via breathalyser, self‐report of recent use and overt signs of intoxication. No exclusions were made based on these procedures. A 14‐panel urine‐drug screen was also administered at session intake. One‐hundred and ninety‐three (*N* = 193) participants completed both diffusion imaging runs and met group criteria (*n*_MA+_ = 95, *n*_MA−_ = 98).

### 1.2 Volumes of Interest

Neurosynth metanalyses were used to define grey‐matter volumes of interest from open, task‐fMRI and human microarray gene‐expression data [26]. One seed and two target volumes were created for diffusion tractography. The two corticolimbic target volumes were also used for task fMRI analyses. Data from the seed volume were not used in those analyses due to restricted fields of view for some participants' fMRI data.

#### 1.2.1 Reward‐Sensitive Targets

**FIGURE 1:**
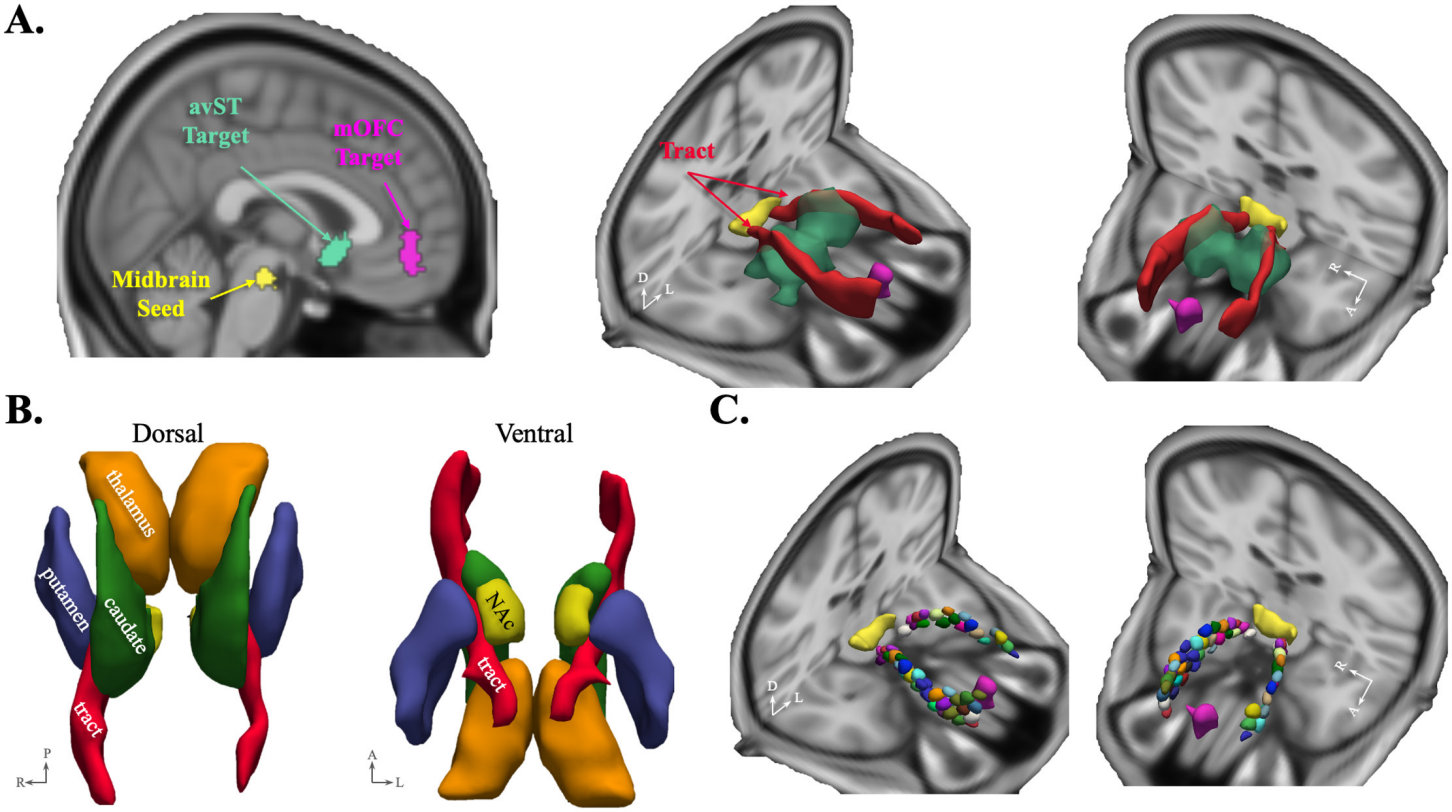
Volumes of interest and the recovered mesocorticolimbic dopamine‐reward tract. (A) *Left:* Sagittal view of the midbrain presynaptic dopamine seed and the two reward‐sensitive, corticolimbic target volumes. *Centre and right*: 3D‐renderings demonstrating the recovered tract. (B) 3D‐renderings of the recovered tract and its path between anatomically defined grey‐matter regions from *Freesurfer's aseg* atlas (see Figures S5–S8 for detailed anatomical context). (C) 3D‐renderings of the 62 discrete, along‐tract nodes as well as the midbrain seed and mOFC target volume. Midsagittal volume image was smoothed; renderings were dilated and smoothed for display. Midbrain seed = midbrain presynaptic dopamine seed; avST Target = reward‐sensitive anterior‐ventral striatum target volume; mOFC Target = reward‐sensitive medial orbitofrontal cortex target volume; Tract = recovered mesocorticolimbic dopamine‐reward tract; NAc = nucleus accumbens.

These were identified via Neurosynth's association test using the search term ‘reward’ [26]. This compared brain activations from 922 studies of reward versus any other term in its corpus. The default, whole‐brain association‐test map (FDR‐corrected *p* < 0.01) was thresholded further (*z* ≥ 6; *k* ≥ 100 contiguous voxels), yielding three voxel clusters of adequate volume that were highly sensitive and reliably responsive to reward stimuli or related processes (see Figure 1A and Table 1). The resultant anterior‐ventral striatum (avST) and medial orbitofrontal cortex (mOFC) voxel clusters were used as reward‐sensitive, corticolimbic target volumes. The third volume, a reward‐sensitive midbrain cluster, was intersected with metanalyses on gene expressions to define the seed volume (described below).

**TABLE 1:** Seed and target volumes of interest.

Volume label	Coordinates			Voxel count	Metanalysis mean *z*‐score			
	*x*	*y*	*z*		Reward	TH	DDC	VMAT2
Anterior‐ventral striatum target	−01	−10	−06	2702	11.35	—	—	—
Medial orbitofrontal cortex target	−03	−46	−07	118	06.64	—	—	—
Presynaptic dopamine midbrain seed	−02	20	−17	137	08.20	02.14	02.00	02.39

*Note:* Coordinates reflect centre of mass of voxel cluster in MNI space (RAS orientation). Metanalysis mean z‐scores derived from Neurosynth ‘reward’ association maps and the Allen Institute's gene expressions.

Abbreviations: DDC = DOPA decarboxylase; TH = tyrosine hydroxylase; VMAT2 = vesicular monoamine transporter (*SLC18A2*).

#### 1.2.2 Midbrain Presynaptic Dopamine Seed

Synthesis and presynaptic release of dopamine from midbrain neurons requires tyrosine hydroxylase (TH) and DOPA decarboxylase (DDC) enzymes, as well as vesicular monoamine transporter protein (VMAT2) [34, 35]. Therefore, we identified voxels highly expressive of TH, DDC and VMAT2 (*SLC18A2*) genes to define *presynaptic* dopamine hubs. The Neurosynth platform performed metanalyses on mRNA transcripts for the three genes using the Allen Institute's human microarray database, providing expression intensity maps for each (normalized *z‐*scores) [36]. A threshold (*z* ≥ 1.5, *k* ≥ 50 contiguous voxels) was applied to each of these maps to provide sufficient volumes of high‐expression voxels (see Figure S1). Spatial overlap among the thresholded maps was then used to identify voxels wherein all three genes were highly expressed (i.e., colocalized) [34, 35]. To enhance functional relevance, only those voxels also overlapping with voxels in the reward‐sensitive midbrain cluster were retained. These procedures produced a volume in which every voxel (1) was within the midbrain, (2) was enriched with all three necessary signatures of presynaptic dopamine neurons and (3) was shown to be sensitive and reliably responsive to rewards (see Figures 1 and S1 and Table 1).

### 1.3 Incentive Processing Task

The Incentive Processing Task (IPT) was adapted from the Human Connectome Project (see Supporting Information) [37, 38]. Participants were practiced on the task and informed that their responses result in winning/losing actual money. Unbeknownst to participants, feedback/trial outcomes were predetermined. Feedback conditions were pseudorandomized into 28 s blocks of mostly reward or mostly loss trials (i.e., Reward and Loss blocks), which standardized task experience and compensation. Participants completed two runs, totalling four blocks (2 × Reward; 2 × Loss; 5 m 44 s total).

### 1.4 Image Acquisition and Processing

Comprehensive details regarding acquisition parameters and image processing are provided in Supporting Information.

#### 1.4.1 Acquisition

Images were acquired via a Siemens 3‐Tesla Skyra with a 32‐channel headcoil. Whole‐brain structural images (1.0 mm^3^ isotropic voxel) were acquired. Diffusion‐weighted images were acquired across two runs (*b* = 1000 s/mm^2^, 64 directions/shell, 1.72 × 1.72 × 2.40 mm^3^ voxel). Two task‐fMRI runs (TR = 1000 ms, 2.5 mm^3^ isotropic voxel) were acquired to recover whole‐brain blood‐oxygen‐level‐dependent (BOLD) signal changes during the IPT. Phase encoding was reversed (AP‐PA) between each diffusion and task‐fMRI run.

#### 1.4.2 Structural and Task fMRI Processing

A standardized, *fmriprep* [39] (*v23.1.2*) preprocessing workflow was used, including bias‐field corrections, brain extraction, nonlinear spatial normalization to a standard template, compartment segmentation (i.e., grey/white matter, cerebrospinal fluid) and motion‐correction and quantification via affine transformations. Vectors for motion censoring were obtained using an a priori threshold (framewise displacement > 0.7) [28], and functional volumes were spatially smoothed at 6‐mm FWHM. Generalized linear models estimated activations during the IPT conditions while controlling for affine transformations, framewise displacements, motion‐censored volumes and other temporal trends (see Supporting Information). These provided beta weights for Reward and Loss blocks. Participant‐level Reward, Loss and Reward > Loss activations were obtained by averaging beta weights across grey‐matter voxels within each target volume.

#### 1.4.3 Diffusion Processing and Tractography

Diffusion processing included: component‐based denoising, brain extraction/masking, estimation of off‐resonance fields across runs, eddy correction with slice‐to‐volume motion correction and susceptibility‐induced distortion correction as well as outlier replacement (see Supporting Information). Fibre orientation distributions were estimated locally using Bayesian models accounting for crossing fibres [40]. Probabilistic tractography reconstructed likely fibre paths connecting the seed to target volumes while accounting for crossing fibres (see Supporting Information) [41]. A streamline density threshold (> 0.01%) was then applied to initially remove unreliable voxels/improbable fibre paths, followed by axial spatial smoothing (4‐mm FWHM)—due to the targeted fibres running predominantly in this plane (see Figure S3) [13]. A final streamline density threshold [42] (> 0.10%) was applied, creating binary participant‐level tract maps used in subsequent, sample‐wide tract recovery.

#### 1.4.4 Tract Recovery and Parcellation

Binary tract maps reconstructed at the participant‐level were aggregated independently for each group. Consistency thresholding was used such that only voxels reconstructed across a majority (> 50%) of group members with recovered streamlines were retained [39]. To ensure that the final tract was not biased by group, an additional consistency criterion was applied such that only voxels that were reproducible across both groups were retained (see Figure S2).

For along tract analysis, the recovered tract was parcellated into spheroids (1.5 × voxel radii max) spaced by at least two voxels on centre, resulting in 62 non‐overlapping nodes (see Figure 1C; see Supporting Information). Node distances along the tract were determined for the left and right sides of the tract independently. Therein, a node's distance from the seed and its inverse distance to mOFC target were calculated, providing a distance‐from‐seed metric that increased along an anterior gradient. For each node, average FA was computed across all voxels contained within it, yielding the 62 FA values per participant used in along‐tract analyses.

### 1.5 Quality Assurance

Quality assurance procedures excluded three MA^+^ for irreconcilable image artefacts (*N* = 190; see Table 2 for characteristics of retained participants). Task‐fMRI data alone were discarded from 5 MA^−^ and 14 MA^+^ due to missing IPT runs, excessive motion (≥ 30% of their functional volumes censored for motion) and/or failure to respond to at least 70% IPT trials [38].

**TABLE 2:** Characteristics of retained sample by group.

	MA^+^	MA^−^	Statistic
*N*	92	98	
Age (mean/range)	41.68 (21–60)	39.36 (19–60)	*t* = 1.51^NS^
Sex (M:F)	49:43	46:52	$\chi^{2}$ = 0.76^NS^
Race (% endorsing)			$\chi^{2}$ = 0.79^NS^ ^a^
Asian/Pacific Islander	00.00	06.19	
Amer. Indian/Alaska Native	07.61	02.06	
Black/African Amer.	11.96	04.12	
Caucasian	70.65	79.38	
Multiple	05.44	08.25	
Unknown	04.35	00.00	
Ethnicity (% endorsing)			$\chi^{2}$ = 1.48^NS^ ^b^
Hispanic/Latine	05.44	11.22	
Not Hispanic/Latine	81.52	86.74	
Unknown	13.04	02.04	
Psych. Rx status (% endorsing)			
Any	25.00	28.57	$\chi^{2}$ = 0.31^NS^
Antidepressant	11.98	20.41	$\chi^{2}$ = 2.52^NS^
Stimulant	04.35	09.18	$\chi^{2}$ = 1.79^NS^
Psych. symptoms (mean/SEM)			
Gen anxiety (GAD‐7)	09.83 (0.68)	04.39 (0.48)	*t* = 6.59***
Major dep. (PHQ‐9)	10.69 (0.74)	04.10 (0.42)	*t* = 7.73***
PTSD (PCL‐5)	31.90 (2.17)	12.61 (1.31)	*t* = 7.61***
Alcohol drink/day (mean/SEM)	01.31 (0.28)	00.33 (0.07)	*t* = 3.36***
Methamphetamine			
Lifetime use (% endorsing)	100.0	—	—
Any 30‐day use (% endorsing)	86.98	—	—
Use‐days, in past 30 (mean/SEM)	18.52 (1.14)	—	—
Freq./day, in past 30 (Mean/SEM)	02.88 (0.43)	—	—
Urine drug screen (% positive)	78.23	—	—

*Note:* Measures and additional characteristics are detailed in Supporting Information.

Abbreviations: F = female; GAD‐7 = Generalized Anxiety Disorder Scale; M = male; MA^+^ = problematic methamphetamine‐use experience group; MA^−^ = methamphetamine‐naïve control group; PCL‐5 = PTSD Checklist for DSM‐5; PHQ‐9 = Patient Health Questionnaire.

^a^ Tests performed on reduced groupings due to limited class samples: Caucasian vs. Not Caucasian/Multiple.

^b^ Tests performed on reduced groupings due to limited class samples: Hispanic/Latine vs. Not Hispanic/Latine.

NS = *p* > 0.05; *p < 0.05; ***p ≤ 0.001.

### 1.6 Hedonic Capacity and Trait Reward‐Responsiveness Reports

Two valid and reliable self‐report measures were used to assess hedonic capacity and tendencies for anticipatory/consummatory arousal from rewarding or pleasant experiences. Importantly, these queried participants regarding nondrug scenarios. Hedonic capacity was assessed via a self‐report Snaith‐Hamilton Pleasure Scale (SHAPS) [43]. SHAPS total scores were used, with lower scores indicating a participant anticipated that they would derive less pleasure from rewards or typically pleasant experiences (e.g., *enjoying favourite meal*, *receiving praise*). The BAS‐Reward Responsiveness subscale of the Behavioral Activation–Behavioral Inhibition Scale (BIS‐BAS) [44] was used to assess trait reward responsiveness. This subscale queried tendencies for positive arousal (e.g., *excited*, *energized*) in anticipation or receipt of rewards (e.g., *winning a contest*) or typically pleasant experiences (see Supporting Information for selection rationale). Lower scores indicated a tendency for lesser (positive) arousal when anticipating or consuming nondrug rewards or pleasant experiences. Other BIS‐BAS subscales are described in Supporting Information.

### 1.7 Analytic Approach

#### 1.7.1 Along Tract Trajectory

Mixed‐effect regression determined FA along the recovered mesocorticolimbic dopamine‐reward tract's trajectory. Linear and polynomial node distance‐from‐seed effects on FA were modelled (i.e., Distance, Distance^2^, Distance × Distance^2^) consistent with anticipated, higher order along‐tract changes in FA [9, 14]. Intercept variability was controlled (random effects of Participant ID), as were differences in total distances and node counts between the left‐ and right‐hemisphere tracts (repeated‐effects of Hemisphere assuming unequal variances). Additional nuisance covariates included fixed‐effects for Head Motion censoring (diffusion outlier volumes; MA^+^ > MA^−^, *p* < 0.05), Age and the Age × Distance interaction (see Supporting Information). Interactions between Group and distance‐from‐seed on FA served as omnibus tests for Group along‐tract differences. Node‐wise tests described approximately where along the tract's trajectory Group effects emerged using median tests (some non‐Gaussian distributions) with an FDR‐corrected *p* < 0.001 threshold on the mixed‐effect model's predicted FA ($\hat{\mathrm{FA}}$).

#### 1.7.2 Localizing Along Tract Group Differences

To localize Group effects, the recovered dopamine‐reward tract was segmented into four discrete bundles based upon surrounding anatomy and distance‐from‐seed [45]. These comprised a Midbrain Termination Bundle, an ALIC Bundle, an Extrastriatal Bundle and an mOFC Termination Bundle (see Supporting Information). Repeated‐measures ANCOVA was used to confirm that FA fluctuated across the four segments as well as localize Group effects. Like the mixed‐effects models, Head Motion and Age were covaried. Bias‐corrected and accelerated bootstrap 95% confidence‐interval tests (B = 1000) evaluated whether Groups exhibited significant differences in marginal‐FA means across bundles.

#### 1.7.3 IPT Activations

The Reward > Loss contrast was used to test Group effects, as this provided differential activations for the Reward condition by removing effects associated with the general task experience (e.g., visual/perceptual load, button pressing) [38]. Groups did not significantly differ in Loss activations (*p*s > 0.05; see Supporting Information). Least‐squares regressions tested whether MA^+^ exhibited reduced Reward > Loss activations relative to MA^−^ while controlling for Head Motion estimates (censored volume counts), which were greater for MA^+^ (*p* < 0.05; see Supporting Information). Individual differences in reward activations were assessed using Reward blocks alone, as individual, but not group, effects can be unduly restricted when using fMRI condition‐condition contrasts (e.g., Reward > Loss) [46].

#### 1.7.4 Functional and Behavioural Associations With Altered Microstructure

Group differences in FA were localized to two tract bundles. We sought to determine whether FA in those altered bundles was related to MA^+^ participants' IPT Reward activations as well as their hedonic capacity and trait reward‐responsivity scores. Least‐squares regressions tested whether FA within those bundles predicted MA^+^ participants' (1) Reward activations within the corticolimbic volumes as well as (2) SHAPS and BAS‐Reward Responsiveness scores. Consistent with Group analyses, effects of Head Motion and Age on FA and the effect of Head Motion on IPT Reward Activations were controlled.

### 1.8 Potential Confounding Factors

Additional testing examined whether potential confounds influenced primary between‐ and within‐group significant effects. This was evaluated by individually entering covariates into the statistical models [28]. Covariates are detailed in Supporting Information. Four covariates were evaluated for between‐groups effects on SHAPS and BAS‐Reward Responsiveness scores, IPT activations and FA across tract bundles. These included psychiatric dimensions (anxious, depressive and PTSD symptoms) and a substance‐use variable (alcoholic drinks per day). These were selected because they (1) could conceivably influence the dependent variables, (2) were significantly different between groups (see Table 2) and (3) were distributed sufficiently within groups to permit the analyses. These four and 12 additional covariates (totalling 16) were evaluated for the within‐MA^+^ group effects to also account for differences in recent MA and other substance use (e.g., past 30‐day use, urine drug‐screen status) as well as psychosis symptom histories.

## 2 Results

### 2.1 Along Mesocorticolimbic Dopamine‐Reward Tract

**FIGURE 2:**
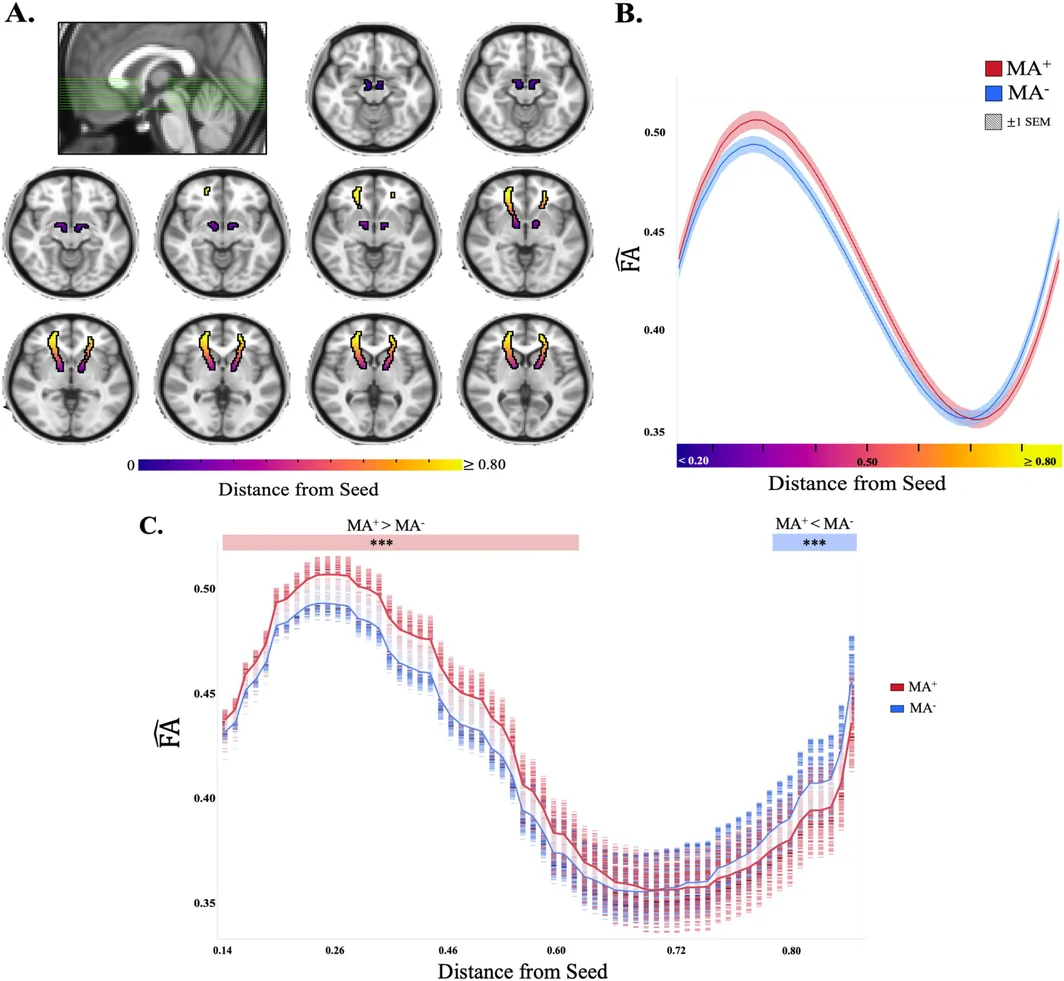
Microstructural coherence along the recovered mesocorticolimbic dopamine‐reward tract. (A) Axial slices illustrate distance‐from‐seed metric (a.u.) increasing along an anterior gradient of the recovered tract. (B) Mixed‐effect model predicted fractional anisotropy (FA) as a function of along tract distance‐from‐seed. Lines show FA trends predicted for each Group, hatched contours indicate ±1 SE of prediction. Heatmap added to x‐axis for parity with Panel (A). (C) Model predicted FA for participant at each of the 62 nodes. Unsmoothed trend lines connect medians across nodes for each Group. Shaded areas between curves illustrate between‐group median differences. MA^+^ = problematic methamphetamine‐use experience group; MA^−^ = methamphetamine‐naïve control group. ***p < 0.001 FDR‐corrected for the 62 between‐group median tests.

Age, Distance and Distance^2^ as well as Age × Distance and Distance × Distance^2^ interactions were significant predictors of FA (*p*s < 0.05; see Supporting Information). Critically, the model did not overfit the data as similar polynomial relationships between distance‐from‐seed and FA were observed in both Groups without mixed‐effects modelling (see Figures 2B and S9). While Group was not a significant predictor of FA (β *=* − 0.003, *p* = 0.108), Group interactions with Distance (β *=* − 0.059, *p* = 0.008) and Distance^2^ (β *=* 0.074, *p* < 0.001) were significant. Post hoc tests indicated increased FA for MA^+^ near posterior portions along the tract (*p*_FDR_ < 0.001; see Figure 2C). Conversely, lower FA values were observed for MA^+^ within white‐matter closest to the mOFC target volume (*p*_FDR_ < 0.001).

### 2.2 Localized FA Alterations Along Tract

**FIGURE 3:**
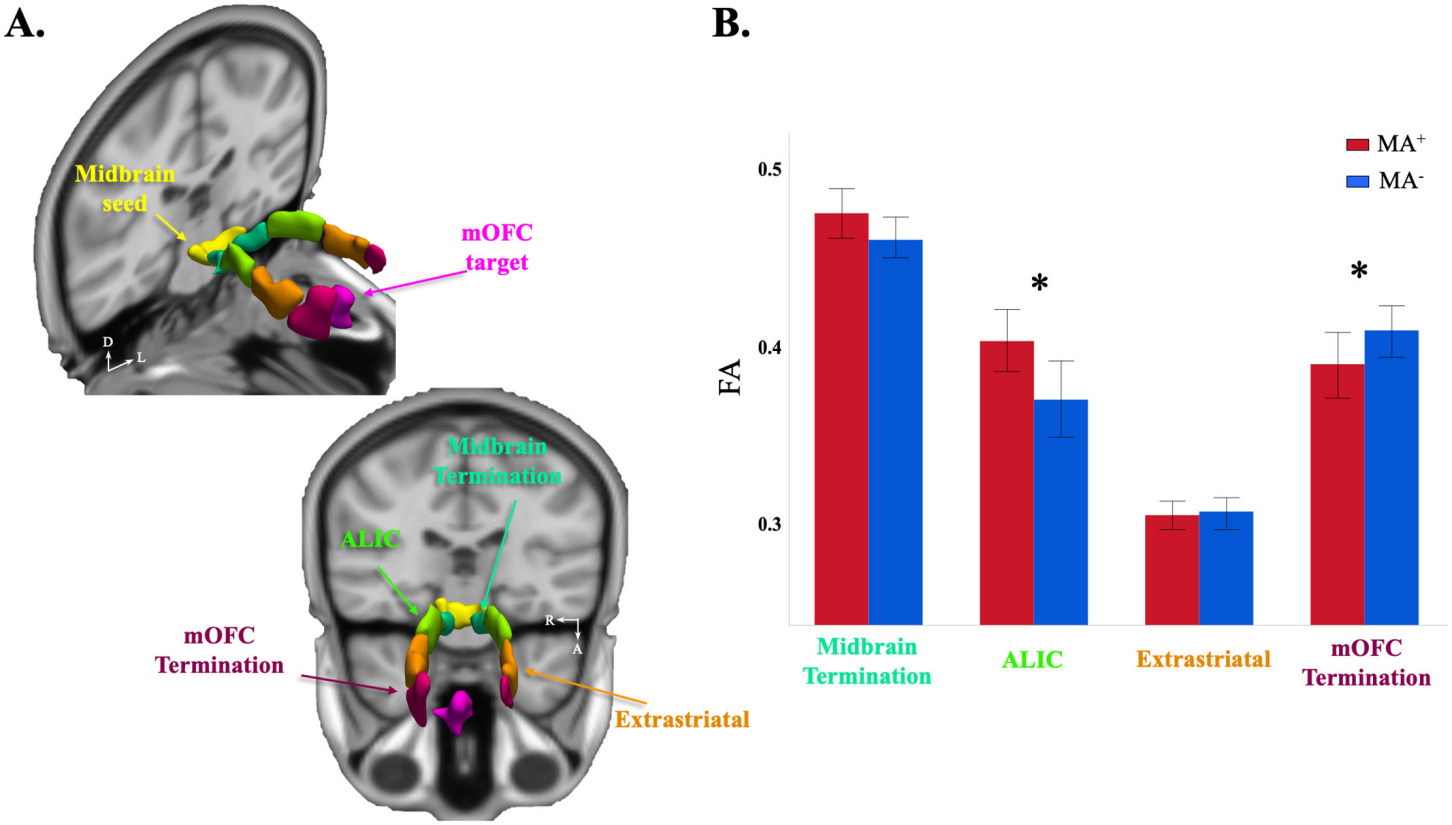
Group effects on microstructural coherence localized within tract bundles. (A) 3D‐renderings of the four bundle segments within the recovered mesocorticolimbic dopamine‐reward tract, dilated and smoothed for display. Gaps were placed between bundle margins to ensure their spatial independence (see Supporting Information). (B) Marginal mean fractional anisotropy (FA) from Group × Bundle repeated‐measures ANCOVA. Midbrain seed = presynaptic dopamine midbrain seed; mOFC Target = reward‐sensitive medial orbitofrontal cortex target volume; ALIC = anterior limb of the internal capsule bundle. MA^+^ = problematic methamphetamine‐use experience group; MA^−^ = methamphetamine‐naïve control group. *p < 0.05 for bootstrapped 95% CI tests on marginal means.

Significant FA fluctuations were confirmed across the four bundle segments and for both groups (*p*s < 0.001; see Supporting Information). Like the mixed‐effect model, the Group main effect was not significant (*F* = 1.33, *p* = 0.250, $\eta_{p}^{2}$ = 0.007) while the Bundle × Group interaction was (*F* = 4.16, *p* = 0.006, $\eta_{p}^{2}$= 0.022). Entering three of the four covariates into the model failed to alter the significant Bundle × Group effect (retained‐effects range: $\eta_{p}^{2}$= 0.017–0.021, *p* = 0.010–0.030). The depressive symptoms covariate weakened the interaction (retained effect: $\eta_{p}^{2}$= 0.012, *p* = 0.083). Significant Group effects on ALIC and mOFC Termination Bundle FA were observed—with MA^+^ exhibiting increased FA within the ALIC Bundle and decreased FA within the mOFC Termination Bundle (*p*s < 0.05; see Figure 3).

### 2.3 Group Effects on Corticolimbic Function: Reward > Loss Activation

**FIGURE 4:**
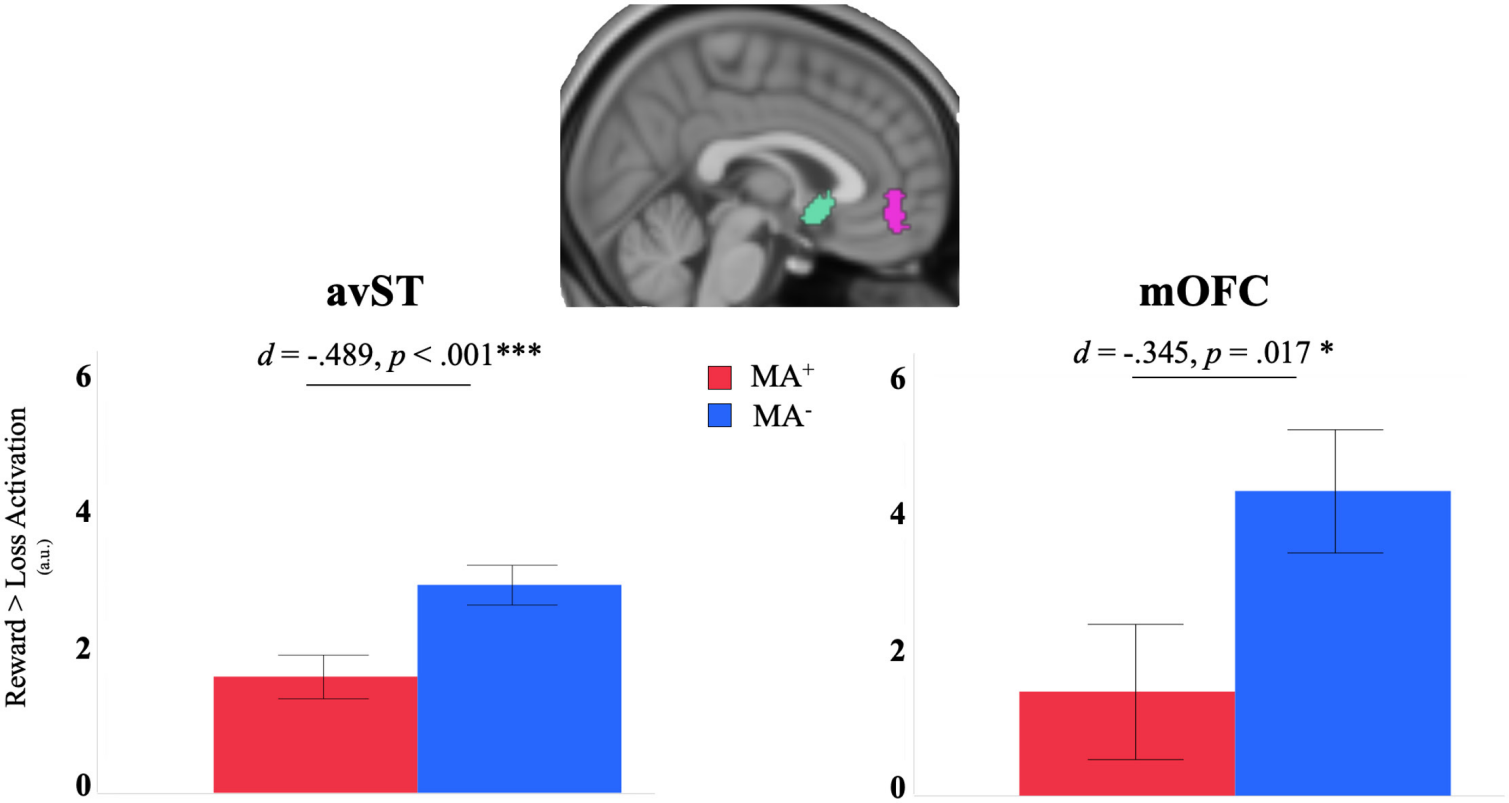
Group effects on Reward > Loss contrast within corticolimbic target volumes. Sagittal view of reward‐sensitive, corticolimbic target volumes; smoothed for display. avST = reward‐sensitive anterior‐ventral striatum target volume (*teal*); mOFC = reward‐sensitive medial orbitofrontal cortex target volume (*fuchsia*). MA^+^ = problematic methamphetamine‐use experience group; MA^−^ = methamphetamine‐naïve control group. *** = *p* < 0.001 and * = *p* < 0.05 for Group means. *d* = Cohen's *d* effect size.

Consistent with our directional hypothesis, planned one‐tailed tests indicated that MA^+^ exhibited reduced activation to the Reward > Loss contrast within the reward‐sensitive avST and mOFC volumes relative to MA^−^ (*p*s_one‐tailed_ < 0.05; see Figure 4). Covariates were not found to alter the significance of Group effects on avST or mOFC activations (retained‐effects range: $\eta_{p}^{2}$ = 0.030–0.076, *p* ≤ 0.001–0.026).

### 2.4 Microstructure‐Function: Localized FA Alterations and MA^+^ Corticolimbic Reward Activations

FA within the ALIC Bundle was significantly associated with MA^+^ participants' Reward activations in the mOFC target volume (β *=* 3.13, *p* = 0.026, *r*_X.Y|Z_ = 0.252). This retained significance controlling for 15 of the 16 within‐group covariates (retained‐effects range: *r*_X.Y|Z_ = 0.219–0.276, *p* = 0.016–0.054), with one covariate (past 30‐day prescription opioid misuse status) weakening effects (retained‐effect: *r*_X.Y|Z_ = 0.203, *p* = 0.082). No other significant microstructure‐function associations were observed (β = −0.34 to 0.271, *p* = 0.656–0.979).

### 2.5 Group Effects on Behavioural Reports

MA^+^ reported significantly lower SHAPS scores than MA^−^, *t* = −5.45, *p* < 0.001, *d* = −0.800. They also reported significantly lower BAS‐Reward Responsiveness scores than MA^−^, *t* = −2.86, *p* = 0.005, *d* = −0.422. None of the four between‐group covariates altered the significance of the Group effects on behavioural reports (retained‐effects range: $\eta_{p}^{2}$ = 0.036–0.119, *p*s ≤ 0.001–0.010).

### 2.6 Microstructure‐Behaviour: Localized FA Alterations and MA^+^ Behavioural Reports

**FIGURE 5:**
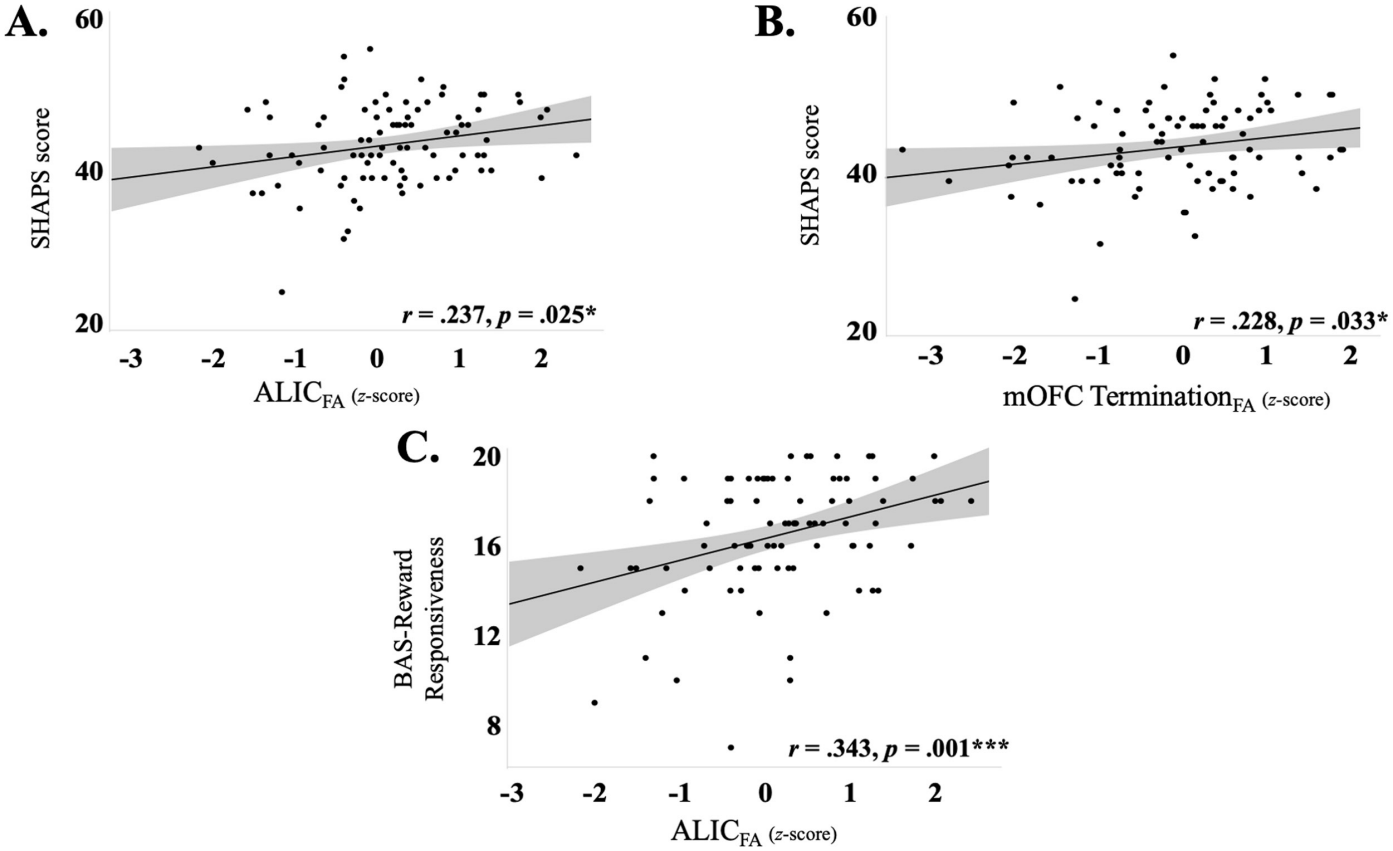
MA^+^ microstructural coherence, SHAPS and reward responsiveness scores. (A and B) MA^+^ participants exhibiting lesser FA within the ALIC Bundle or mOFC Termination Bundle reported lower SHAPS scores than their peers exhibiting greater FA. (C) MA^+^ participants exhibiting lesser FA within the ALIC Bundle reported lower BAS‐Reward Responsiveness scores than their peers exhibiting greater FA. MA^+^ = problematic methamphetamine‐use experience group; FA = fractional anisotropy; ALIC = Anterior Limb of the Internal Capsule Bundle; mOFC Termination = Medial Orbitofrontal Cortex Termination Bundle. SHAPS = Snaith‐Hamilton Pleasure scale; BAS‐Reward Responsiveness = Reward responsiveness subscale. FA values reflect standardized residuals controlling for age and head motion effects. ***p < 0.001; *p < 0.05.

FA within ALIC and mOFC Termination Bundles was significantly associated with SHAPS scores (*p*s < 0.05) such that MA^+^ participants who exhibited lesser FA within also reported lower SHAPS scores (see Figure 5A,B). A similar association was observed between BAS‐Reward Responsiveness scores and FA within the ALIC Bundle (*p* < 0.05; see Figure 5C). None of the 16 within‐group covariates altered the significance of any of these associations (retained‐effects range: *r*_X.Y|Z_ = 0.209–0.355, *p*s ≤ 0.001–0.052). A significant association was not observed for FA within the mOFC Termination Bundle and BAS‐Reward Responsiveness (β *=* 0.424, *p* = 0.115).

## 3 Discussion

We sought to characterize microstructural alterations along a white‐matter tract plausibly supporting reward signalling between midbrain dopamine hubs, striatum and orbitofrontal cortex among people with problematic MA‐use experience. MA^+^ exhibited microstructural alterations along this mesocorticolimbic dopamine‐reward tract, as well as neurofunctional and behavioural deficits concerning nondrug rewards. Associations between microstructural coherence within tract bundles that were altered among MA^+^ participants and their grey‐matter responses to nondrug rewards yielded mixed results. Specifically, the only significant association observed was that between microstructural coherence within the ALIC and reward responses within mOFC. However, within those tract bundles, microstructural coherence was significantly associated with MA^+^ participants' behavioural reports of hedonic capacity and trait reward responsiveness.

MA^+^ participants exhibited alterations along the mesocorticolimbic dopamine‐reward tract, including both increases and decreases in microstructural coherence compared to MA^−^. Increases were localized to a tract bundle traversing the ALIC. Decreases were localized to the mOFC Termination Bundle. This complex pattern, coupled with the inexact nature of FA, requires cautious interpretation. This is especially necessary regarding the ALIC, which is a dense waypoint for subcortical and infratentorial tracts. Additionally, FA within the ALIC is acutely sensitive to inflammatory processes, which have been associated with methamphetamine use [47, 48]. Despite interpretive challenges, some speculation regarding these findings is possible. To wit, the microstructural alterations observed within the ALIC could indicate stronger white‐matter connections between midbrain dopamine hubs and avST regions for MA^+^ participants relative to their peers. In contrast, their white‐matter connections between these hubs and mOFC may be relatively weak.

Although our interpretation should be considered as tentative until the cellular mechanisms are confirmed, the pattern of MA^+^ participants' mesocorticolimbic tract alterations appears consistent with neuroadaptations posited by contemporary addiction theories [1, 2, 3, 4]. Specifically, MA^+^ participants' increased microstructural coherence in the ALIC Bundle could reflect adaptations from chronic hyperactivation of this pathway via MA. Indeed, recent preclinical work investigating other drugs of abuse has found increases in myelination (via oligodendrogenesis) of midbrain dopaminergic axons projecting to nucleus accumbens following single and chronic exposures [49]. Moreover, increased functional connectivity between midbrain and striatum has been found in preclinical models of MA addiction and people with MA use disorder [10, 50]. On the other hand, MA^+^ participants' relatively decreased microstructural coherence in the mOFC Terminal Bundle may follow from a compensatory downregulation of prefrontal glutamatergic transmission associated with MA use [1]. Deficits in prefrontal glutamate observed among people with MA use disorder and decreased functional connectivity between medial prefrontal cortex and midbrain observed in preclinical models of MA addiction lend support for this idea [10, 51]. Downregulation of glutamatergic signalling would also be expected to limit the functioning or weaken the microstructures that support white‐matter connections and neurotransmission [52, 53]. It is worth emphasizing, however, that a link between prefrontal glutamate downregulation and weaker structural connectivity still requires confirmation in people who use MA.

Neurofunctional and behavioural deficits observed among MA^+^ participants were congruent with phenomena of the ‘dark side’ of addiction—characterized by diminished valuation and the loss of motivational salience of nondrug rewards [2, 4]. Specifically, compared to MA^−^, MA^+^ participants' corticolimbic grey‐matter responses were hyposensitive to monetary rewards. Their hedonic capacity and trait reward responsiveness scores were also significantly lower than MA^−^. These reports assessed subjective valuation and the motivational salience of imagined nondrug rewards/pleasant experiences. Thus, findings may have arisen from a deficit in generating such mental representations. However, this explanation is difficult to reconcile with extant findings among people experienced with stimulants [54]. A more plausible explanation, that is also consistent with human and preclinical findings [7, 18, 19], is that the prospect of nondrug rewards or experiences offered relatively low hedonic value and evinced little motivational salience for MA^+^.

Alterations within mesocorticolimbic circuits are theorized to play a central role in the low hedonic capacity and diminished motivation to pursue typical (nondrug) activities observed in addiction [2, 4]. Our findings provided evidence to newly support those claims for human MA addiction. Significant associations were observed between MA^+^ participants' behavioural reports and microstructural coherence within the dopamine‐reward tract bundles that showed group differences (i.e., those that were altered for MA^+^). Specifically, MA^+^ participants exhibiting decreased FA within either the ALIC or mOFC Termination Bundles reported lower hedonic capacity relative to their peers. A similar relationship was observed between FA within the ALIC Bundle and trait reward responsiveness. It remains unclear which factor drove the microstructure‐behaviour relationships. However, findings indicating a causal role for mesocorticolimbic circuitry in similar behaviours favour the possibility of mesocorticolimbic influence [20, 21]. Further, while evidence is limited regarding MA, a causal role for mesocorticolimbic circuitry in modulating hedonic capacity and motivational salience has been established among people treated for other substance use disorders via ALIC and/or accumbenal deep‐brain stimulation [55, 56].

### 3.1 Limitations and Future Directions

Our findings should be considered in the context of several limitations. First, we recruited from a community cohort and did not exclude polysubstance use or psychiatric comorbidities. This was intentional in seeking to capture characteristics of the broader population of people who use MA [57]—for whom residential treatment is unlikely and polysubstance/psychiatric issues are common [32, 33]. Our findings were largely unaffected by putative confounds related to those design choices (e.g., positive drug screens, psychiatric symptoms). However, residential settings and samples without polysubstance/psychiatric comorbidities may be prudent alternatives for future replications. Second, group differences were observed in psychiatric symptoms and head motion. All but one of those factors (i.e., depressive symptoms on Bundle × Group interaction) failed to impact the significance of primary results. Follow‐up testing further showed that associations between FA and MA^+^ participants' depressive symptoms failed to reach significance (see Supporting Information). Nevertheless, high rates of major depressive disorder among people with MA use disorder along with mechanistic links between mesocorticolimbic circuits and major depressive disorder highlight the need for future studies more focused on disentangling the role that comorbid major depressive disorder could play in the observed mesocorticolimbic tract alterations [20, 21, 22, 58]. Third, we examined FA, which afforded a composite index of white‐matter microstructures. More advanced approaches are still needed to disentangle the cellular bases of the current findings (e.g., myelin volume). Fourth, tractography reconstructed likely fibre paths. These were reproducible across groups, demonstrated specificity by way of minimal spatial overlap with proximal tracts (0%–25%; see Supporting Information) and, corresponded with ground‐truth human anatomical findings (see Supporting Information) [13]. However, the recovered tract's topology should be considered a plausible (but coarse) approximation of corticolimbic structural connections with midbrain presynaptic dopamine hubs until confirmed through more precise methods (e.g., microdissection). Finally, our analyses were cross‐sectional and correlational. Therefore, research is needed to adjudicate whether the observed effects were the consequences of MA use or whether these existed prior.

## 4 Conclusions

Our findings bridged a critical gap between neurobiological theory and evidence in MA addiction by identifying alterations within a mesocorticolimbic dopamine‐reward tract among people with problematic MA‐use experience. Notably, the microstructural alterations observed within this tract were consistent with adaptations within mesocorticolimbic circuits posited by several neurobiological theories of addiction [1, 2, 3, 4]. Mesocorticolimbic microstructure and grey‐matter function associations were limited. However, microstructural coherence in altered dopamine‐reward tract bundles was significantly related to MA^+^ participants' attributions of hedonic value and motivational salience to nondrug rewards. With treatments for other addictions targeting the modulation of dopamine and reward systems [54, 55, 56], the findings provided here may afford a foothold for future implementations of these treatments or their optimization in people MA use disorder.

## Author Contributions

R.J.R.: data collection, writing, revisions. K.T.W.: writing, revisions. R.J.R.B.: methodology, writing, revisions. N.A.H.: funding, conceptualization, supervision, analysis, methodology, project administration, writing.

## Funding

This project was supported by the Rural Drug Addiction Research Center at the University of Nebraska‐Lincoln.

## Ethics Statement

Study procedures were approved by the University of Nebraska–Lincoln Institutional Review Board, and informed consent was obtained before study procedures.

## Conflicts of Interest

The authors declare no conflicts of interest.

## Supporting information


**Figure S1:** Midbrain seed creation via gene‐function colocalization.
**Figure S2:** Group‐level tract reconstruction probabilities and final tract.
**Figure S3:** Group‐level primary diffusion directions within tract.
**Figure S4:** Spheroid parcellations contained within tract.
**Figure S5:** Anatomical context of Midbrain Termination Bundle segment.
**Figure S6:** Anatomical context of ALIC Bundle segment.
**Figure S7:** Anatomical context of Extrastriatal Bundle segment.
**Figure S8:** Anatomical context of mOFC Termination Bundle segment.
**Figure S9:** Raw along tract distance and fractional anisotropy scatterplot.
**Figure S10:** Specificity comparison: Anterior thalamic radiations.
**Figure S11:** Specificity comparison: Forceps minor.
**Figure S12:** Specificity comparison: Uncinate fasciculus.
**Table S1:** Detailed MA+ group substance use characteristics.

## Data Availability

Data supporting study findings are available upon reasonable request to the corresponding author.
