# Supplementary material for: Mesocorticolimbic Dopamine‐Reward Tract Alterations and the ‘Dark Side’ of Methamphetamine Addiction
Source: Addict Biol. 2026 Sep 16;31(9):e70186. doi: 10.1111/adb.70186 (PMC13583458; doi:10.1111/adb.70186)
Supplement: Supplementary file 1 [file ADB-31-e70186-s001.docx]

**Supporting Information**

I. Supporting Methods

II. Supporting Results

III. Supporting Figures

a. *Supporting Figure 1. Midbrain Seed Creation via Gene-Function Colocalization*

b. *Supporting Figure 2. Group-level Tract Reconstruction Probabilities & Final Tract*

c. *Supporting Figure 3. Group-level Primary Diffusion Directions within Tract*

d. *Supporting Figure 4. Spheroid Parcellations Contained within Tract*

e. *Supporting Figure 5. Anatomical Context of Midbrain Termination Bundle Segment*

f. *Supporting Figure 6. Anatomical Context of ALIC Bundle Segment*

g. *Supporting Figure 7. Anatomical Context of Extrastriatal Bundle Segment*

h. *Supporting Figure 8. Anatomical Context of mOFC Termination Bundle Segment*

i. *Supporting Figure 9. Raw Along Tract Distance & Fractional Anisotropy Scatterplot*

j. *Supporting Figure 10. Specificity Comparison: Anterior Thalamic Radiations*

k. *Supporting Figure 11. Specificity Comparison: Forceps Minor*

l. *Supporting Figure 12. Specificity Comparison: Uncinate Fasciculus*

IV. Supporting Tables

a. *Supporting Table 1. Detailed MA^+^ Group Substance Use Characteristics*

V. Supporting References

**Supporting Methods**

*Midbrain Presynaptic Dopamine Seed*

Supporting Figure 1 illustrates the definition of the presynaptic dopamine seed volume by colocalizing Allen Human Brain Atlas gene-expression metanalyses (DDC, VMAT2 [*SLC18A2*], TH) and Neurosynth’s automated association test comprising 922 studies of “reward”.^1^

*Incentive Processing Task*

The IPT presented a “?” cue for 1.5s indicating that participants needed to guess (via button press) whether a forthcoming number was greater or less than five. Feedback was provided for 1s immediately after a response, followed by a 1s intertrial interval. For reward trials, feedback was a green arrow pointing upward with “+$1” written beneath. For loss trials, feedback was a red arrow pointing downward with “-$.50” written beneath. For neutral trials (uncommon), feedback was a bidirectional grey arrow with “+$0” written beneath. If no response was provided within the allotted 1.5s window, the feedback screen read “no response”.

*Image Acquisition Details*

**Structural.** Whole-brain anatomical images (160 slices) were acquired via a multiecho, T1w-MPRAGE sequence: TR = 2530ms, TE_1/2/3/4_ = 1.55/3.04/4.53/6.02ms, Flip Angle = 7.0${}^{\circ}$, 1.0mm^3^ isotropic voxel. T1 images were acquired with volumetric navigators for prospective motion correction to increase image fidelity and limit data loss.^2^

**Task-fMRI.** Task-fMRI runs were acquired using multiband gradient-recalled echo-planar sequences for whole-brain (51 axial slices) BOLD signal: TR = 1000ms, TE = 29.80ms, Flip Angle = 60${}^{\circ}$, 2.5mm^3^ isotropic voxel, 124 dynamics/run.

**Diffusion-MRI.** Multiband gradient-recalled echo-planar sequences were used to acquire whole-brain (60 axial slices) diffusion-weighted images: TR = 3000ms, TE = 95.00ms, *b* = 1000 s/mm^2^, 64 directions/shell, 1.72 $\times$ 1.72 $\times$ 2.40 mm^3^ voxel, 134 dynamics/run.

*Image Processing Details*

**Structural & Task fMRI Processing**. Nonlinear spatial normalization warped structural and task-fMRI data to the ICBM-152 Nonlinear Asymmetrical standard template.^3^ AFNI’s *3dDeconvolve*^4^ was used to construct generalized linear models estimating activations during the IPT conditions while (1) controlling for translation and rotation motion parameters, and framewise displacement; (2) preventing volumes deemed motion outliers from influencing task-activation outputs (i.e., censoring) and; (3) employing an automatic polynomial (high-pass) filter to minimize temporal trends (e.g., signal drifts). Within these models, participant-level task activations were obtained by convolving the design matrix and AFNI’s block impulse-response functions, the product of which was regressed onto participants’ BOLD time series’ while controlling for the aforementioned nuisance factors.

**Diffusion Processing.** Data underwent component-based denoising via MRtrix3’s *dwidenoise.*^5^ Following skull-extraction/masking with FSL’s *bet2*, *topup* was used to estimate off-resonance fields across the reversed phase-encoded runs.^6^ Eddy correction was performed via *eddy_cuda* (*v10.2*), which included slice-to-volume motion correction, susceptibility-induced distortion with motion correction, and outlier replacement.^7–10^ Quality assurance metrics were extracted through *eddy_quad* (e.g., motion outlier frames).^11^ Fiber orientation distributions were estimated locally via *bedpostX-gpu* using Bayesian models accounting for crossing fibers along with rotated *b*-vectors/values and gradient nonlinearities.^12^

*Tractography, Parcellation, & Bundle Segmentations*

**Tractography Workflow**. Seed and target volumes were transformed to native spaces. Tracking was constrained within native white-matter boundaries (obtained via *fMRIprep*’s implementation of *recon-all*) and performed using *probtrackx2_gpu*.^13^ Termination masks were used to prevent streamlines propagating into brainstem or anterior to the mOFC target. Exclusion masks prevented reconstructing biologically-implausible fiber paths (e.g., intersecting ventricles or other, non-white matter compartments) and prevented streamline propagation into fibers posterior to the seed volume, as well as superior or lateral to the avST target volume. Other tracking parameters included modified Euler streamlining, 50000 individual path samples, distribution corrections for path length, and allowing streamlines to reach either target volume. *probtrackx2_gpu* defaults were used for: step length = 0.5, number of steps per sample = 2000, curvature threshold = 0.2, loop checking, and subsidiary fiber volume threshold = 0.01.

**Tract Recovery**. Supporting Figure 2 demonstrates group-level reconstruction probabilities of the preliminary tracts. Supporting Figure 2 also illustrates the resulting/final tract recovered from combining (i.e., conjoint probabilities) consistency thresholding across groups. Supporting Figure 3 illustrates the average primary diffusion direction of voxels within the recovered tract for both groups. Both groups exhibited expected anterior-posterior bias within this tract.

**Tract Parcellation.** Spheroid parcellations conferred advantages over other along-tract approaches, including (1) forming discrete nodes in native resolution (e.g., upsampling not required), (2) maintaining the tract’s geometry (i.e., spheroids conformed to tract shape, boundaries, and distance; see Supporting Figure 4), and (3) optimizing node counts and spatial distributions based upon the tract’s complete geometry (i.e., versus arbitrary specification). Potential drawbacks of this approach, including hemispheric differences in node counts (left = 20, right = 42), spacings, and total distances were mitigated via the mixed-effect statistical modeling approach (detailed in Main Text). Node distance-from-seed was calculated using AFNI’s modified Euclidean distance transforms (*3dDepthMap*). A standard tensor model was fit locally to estimate voxel-wise diffusion scaler values*.*^14^

**Bundle Segmentations.** Four bundle segments were generated from the recovered tract based upon relative distance from the seed volume and surrounding anatomy (described below). Gaps were paced between bundle margins to ensure spatial independence (see Main Text Figure 4A). This resulted in retention of 56 of the 62 nodes (90%), which were labeled based upon the bundle segment they resided in, and were used to determine the localized effects described in the Main Text. The surrounding anatomy of the 4 bundle segmentations was characterized (and is described below) using a manually-labeled, high-resolution subcortical atlas.^15^ Additional anatomical labels were provided via *Freesurfer* white-matter parcellations (*wmparc*). The absolute distances from anatomical labels (in mm) described below were calculated using the *Freeview* measure tool and reflect estimates averaged across both bilateral tract segments within the *MNI152-Nonlin-Asym2009* (1mm) template space.

Midbrain Termination Bundle (*d*_along_tract_ < .20). The segment began 22mm posterior to accumbenal inferior margins (see Supporting Figure 5). Its posterior margins were near substantia nigra and 2mm anterior of ventral tegmental area, as well as inferior and anterior to parabrachial pigmented nuclei. Its path remained lateral to hypothalamus and medial to globus pallidus internus, with tract margins also expanding vertically ($\Delta$13mm in-plane peak). Its anterior endpoint ($\Delta$14mm anterior of start point) terminated prior to the emergence of the anterior commissure in the coronal view.

ALIC Bundle (*d*_along_tract_ = .25–.50). The segment began 5mm posterior to accumbenal superior margins (see Supporting Figure 6). Its posterior margins were inferior and posterior to anterior commissure. It projected superior in white matter medial to globus pallida and remained lateral to fornix anterior columns, whilst continuing anteriorly within 1-3mm lateral to accumbens. Its path ascended above anterior commissure into the internal capsule—following documented paths of human mesocorticolimbic fibers.^16^ Therein, tract margins expanded mostly vertically ($\Delta$14mm in-plane peak) prior to its anterior endpoint near caudate head ($\Delta$21mm anterior of start point).

Extrastriatal Bundle (*d*_along_tract_ = .55–.75). The segment began 9mm anterior to accumbenal superior margins (see Supporting Figure 7). Its posterior margins were near anterior aspects of caudate head and emerged immediately prior to the disappearance of putamen in the coronal view. Its margins expanded mostly vertically ($\Delta$19mm in-plane peak) and remained predominantly within unsegmented white-matter territories through its anterior endpoint ($\Delta$22mm anterior of start point).

mOFC Termination Bundle (*d*_along_tract_ $\geq$ .80). The segment began 28mm anterior of accumbenal superior margins (see Supporting Figure 8). Its posterior margins were mostly confined within unsegmented white-matter territories. The segment’s vertical expansion ($\Delta$21mm in-plane peak) throughout its anterior path resulted in intersections with lateral and medial orbitofrontal as well as superior frontal white-matter territories. Its endpoint ($\Delta$13mm anterior of start point) terminated in white matter lateral to the mOFC target volume.

*Along Tract Trajectory: Covariates*

Age and head motion were included as covariates in along-tract analyses. The rationale for including Age as a covariate was based upon this study’s wide age range (19 – 60) and established changes in FA that occur across the lifespan.^17,18^ Head motion was also chosen as a covariate due to significant differences between groups (see Supporting Results) and the established impacts of head motion on diffusion signals.^11^

*BAS-Reward Responsiveness Selection Rationale*

The BAS-Reward Responsiveness subscale was the focus of testing because (1) it has been shown to relate to the perceptual experience of, and neural responses to, drugs of abuse^19^ and, (2) it is unique from other BAS subscales (i.e., BAS-Drive, BAS-Fun Seeking) in that it is largely unrelated to potentially confounding traits, like impulsivity.^20^ Moreover, despite correlations amongst BAS subscales, they are conceptually and empirically distinct as well as predict unique reinforcement phenomena.^19–22^

*Potential Confounding Factors*

**Between-group.** Where indicated in the Main Text, four covariates were evaluated by individually entering them into the target models. These included three reliable and valid measures of participants’ current psychiatric symptoms, comprising: major depression symptoms assessed via the Patient Health Questionnaire 9-item scale (PHQ-9)^23^; generalized anxiety symptoms via the Generalized Anxiety Disorder 7-item scale (GAD-7)^24^; and post-traumatic stress symptoms via the PTSD Checklist for DSM-5 (PCL-5).^25^ The fourth, between-group covariate was the average number of alcoholic beverages consumed per day assessed via the 30-day timeline follow-back paradigm.^26^

**Within-MA^+^ group.** Where indicated in the Main Text, 16 covariates were individually entered into the target models to test whether they influenced the significance of findings among MA^+^ participants. All four between-group covariates (described above) were evaluated in these analyses. An additional 12 covariates were tested to evaluate whether recent substance use or psychosis symptoms may have influenced findings. These 12 additional covariates are described below.

Psychosis Symptoms (1 variable). The extent of lifetime psychosis experiences/symptoms was evaluated via the Psychosis Symptom Assessment Scale, a reliable measure that was designed to isolate substance-induced and organic psychosis symptoms based upon DSM-IV criteria in populations with stimulant-use disorders.^27^ The original version was adapted here to specifically query MA-use episodes which provided: the frequency of psychosis symptoms experienced proximal to recent MA-use episodes (within 24 hrs of last use), those occurring during extended periods of abstinence (greater than one week of abstinence), and the combined frequency of induced and non-induced psychosis symptoms.

Urine Toxicology (3 variables). A urine drug screen (CLIA-Waved INSTI Drug Test®) was administered onsite during the day of the MRI to detect recent use of MA or other common drugs. The 14-panel screen permitted biological confirmation of recent use of the following agents or their metabolites: amphetamines, barbiturates, burprenorphine, benzodiazepines, cocaine, MA, 3,4-methylenedioxymethamphetamine (MDMA/ecstasy), methadone, EDDP, tetrahydrocannabinol (THC), generic opioids, oxycontin, phencyclidine (PCP), and tricyclic antidepressants. MA (78.3% positive) and THC (58.7% positive) were frequently detected; thus, the detection status of each of these substances were evaluated as individual covariates. Amphetamine and MDMA detections were also elevated (>40% positive) but these were not reported nor evaluated as covariates due to their strong tendency for cross-reactivity in the presence of MA.^28^ However, past-month use of these substances was captured via self-report (see *Past 30-day Substance Use Reports*). Detection status of tricyclic antidepressants was not evaluated. A single, binary variable was created to evaluate the detection status of the 9 other aforementioned substances to provide a sufficient number of positive detections for analysis (< 10% positive for any assay). See also Supporting Table 1.

Past 30-day Substance Use Reports (8 variables). Urine screening for most addictive substances can only detect use within 2-7 days.^28^ Thus, we evaluated 30-day substance-use patterns via the Risk Behavior Survey, a reliable and valid measure of recent drug use in community samples.^29,30^ This permitted evaluating binary variables of use within the past 30 days, as well as continuous variables quantifying the frequency of use (per day) within the past 30. Illicit substances queried included cannabis/marijuana, cocaine, MA, hallucinogens (including MDMA), and street opioids (including heroin/opioid derivatives). Misuse of legal substances was also queried including inhalants (e.g., nitrous, glue), as was the non-medical use/misuse of prescription stimulants, sedatives, and opioids. Endorsement of any MA (87.0% endorsing) or cannabis use (67.4% endorsing) within the past 30-days was common. The use-frequencies reported for these substances were also sufficiently distributed to permit evaluation of these as continuous variables. Specifically, the frequency of use episodes per day reported by each participant over the past 30 days was used to create an episode/day frequency composite (Mean_MA_ = 2.88 episodes/day; Mean_cannabis_ = 1.51 episodes/day). Distributions of the episode/day frequencies for all other substances were highly restricted; thus, these were solely evaluated as binary variables. Non-medical/misuse status of prescription opioids (17.4% endorsing), prescription sedatives (16.3% endorsing), and prescription stimulants (11.0% endorsing) was evaluated for each of these substances individually. Use status of hallucinogens (17.4% endorsing) and cocaine (14.1% endorsing) was also evaluated. These and the more rarely endorsed substances (i.e., inhalants and street opioids) were further combined into a single binary variable to evaluate their effects. See also Supporting Table 1.

*Specificity of the Recovered Dopamine-Reward Track*

Specificity of the mesocorticolimbic dopamine-reward tract was evaluated by testing its spatial consistency/independence with three proximal, anterior white-matter tracts (i.e., anterior thalamic radiations [ATR], forceps minor [FMI], and uncinate fasciculi [UF]). Diffusion data underwent the same processing and quality assurance procedures described for the mesocorticolimbic dopamine-reward tract. However, ATR, FMI, and UF were reconstructed using standardized probabilistic tractography protocols via *xtract*.^31^ Fiber tracking proceeded in native space using *xtract* default parameters alongside its validated and standardized seed-, target-, termination-, and exclusion-volumes. Consistent with recovery of the mesocorticolimbic dopamine-reward tract, the native-space streamline density maps were thresholded to create binary participant-level tract maps following reconstruction. To recover these comparison tracts, however, a more liberal density threshold (.05% versus .10%) was used to expand their margins and thus afford a more stringent comparison for the dopamine-reward tract. Participant-level tract maps were then nonlinearly warped to standard space and aggregated separately for each group. The same 50% consistency thresholding procedure was used to recover the final ATR, FMI, and UF tracts as that described in the Main Text. Spatial consistency between those tracts and the recovered mesocorticolimbic dopamine-reward tract was quantified via Dice’s $\kappa$.^32,33^ Spatial independence was quantified using Jaccard Distance, along with an index describing the percentage of voxels within the dopamine-reward tract that were spatially-independent from each comparison tract (D-R Unique Voxels).

**ATR.** Like the recovered mesocorticolimbic dopamine-reward tract, ATR feature projection fibers connecting medial subcortical nuclei to prefrontal cortices that traverse the anterior internal capsule and remain unilateral. ATR, however, connect thalamus to prefrontal cortex/pre-central gyrus.^31^ As such the mesocorticolimbic dopamine-reward tract should proceed in parallel while remaining more inferior and lateral to the recovered ATR projections.^16,34,35^

**FMI.** These commissural fibers connect right and left prefrontal cortices at the genu of corpus callosum. Connections extend superior and anterior from their midline origin.^31^ As the recovered mesocorticolimbic dopamine reward tract does not cross the midline, its margins were expected to proceed lateral to those of the FMI.

**UF.** These association fibers traverse the inferior extreme capsule, connecting anterior temporal and inferior prefrontal cortices.^31^ As such the margins of the recovered dopamine-reward tract should proceed medial to UF, and its prefrontal terminations should be superior UF.^36^

**Supporting Results**

*Group Effects on Head Motion*

The number of diffusion motion outlier frames (*t* = 4.55, *p <* .001) and task-fMRI motion outlier frames (*t* = 3.18, *p* = .002) were significantly greater for MA^+^ than MA^-^. Thus, between-group tests using these images covaried for their respective head motion variables.

*Detailed Along Tract Mixed-Model Effects*

**Random Intercept Effects & Repeated Factors Covariance Estimates**

- Participant ID: $\beta$ = 0.00065, Wald *p* < .001
- Hemisphere Left*:* $\beta$ = 0.0088, *LCL_95%_* = 0.0084, *UCL_95%_* = 0.0092
- Hemisphere Right: $\beta$ = 0.0086, *LCL_95%_* = 0.0083, *UCL_95%_* = 0.0089

**Intercept & Nuisance Covariates Fixed Effects**

- Intercept: $\beta$ = -0.47104, *t* = -15.09, *p* < .002
- Age: $\beta$ = -0.00068, *t* = -3.54, *p* = .005
- Age$\times$Distance: $\beta$ = -0.00114, *t* = -3.05, *p* = .002
- Head Motion: $\beta$ = -0.00740, *t* = -1.31, *p* = .193

**Targeted Fixed Effects**

- Distance: $\beta$ = 3.655, *t* = 31.94, *p* < .001
- Distance^2^: $\beta$ = -3.666, *t* = -33.55, *p* < .001
- Distance$\times$Distance^2^: $\beta$ = 3.903, *t* = 34.86, *p* < .001
- Group: $\beta$ = -0.0035, *t* = -1.62, *p* = .108
- Group$\times$Distance: $\beta$ = -0.05863, *t* = -2.66, *p* = .008
- Group$\times$Distance^2^: $\beta$ = 0.07360, *t* = 3.46, *p* < .001

*Localized FA Alterations Along Tract*

The repeated-measures ANCOVA confirmed significant FA fluctuations across the four bundle segments (*F* = 12.64, *p* < .001, $\eta_{p}^{2}$ = .065). Simple main-effects tests confirmed these effects for both MA^+^ (*F* = 3.58, *p* = .015) and MA^-^ (*F* = 8.27, *p* < .001).

*Group Effects on Function: Loss Activations*

Significant Group effects were not observed for Loss activations within the aVST (*t* = -1.87, *p* = .063) or mOFC volumes (*t* = 0.72, *p* = .474).

*Group Effects on Other BIS-BAS Scales*

Significant group differences were not observed in BAS-Drive (*t* = -0.40, *p* = .689) and BAS-Fun Seeking subscales (*t* = -0.03, *p* = .976). MA^+^ had significantly lower Behavioral Inhibition Scale (BIS) scores compared to MA^-^ (*t* = -2.88, *p* = .004).

*Specificity of the Recovered Dopamine-Reward Track*

**Anterior Thalamic Radiations (ATR).** The mesocorticolimbic dopamine-reward tract was found to be mostly distinct from the ATR (see Supporting Figure 10). The tracts exhibited poor spatial consistency ($\kappa$ **=** .438) and moderate spatial independence (Jaccard Distance = .781), with around three quarters of the dopamine-reward tract’s voxels falling outside of the ATR’s margins (D-R Unique Voxels = 74.86%). Qualitatively, the dopamine-reward tract’s margins also were also observed to proceed more inferior and lateral relative to the ATR (see Supporting Figure 10B).^16,34,36^

**Forceps Minor (FMI).** The dopamine-reward tract was distinct from the FMI (see Supporting Figure 11). The tracts exhibited virtually no spatial consistency ($\kappa$ **=** .056) and strong independence (Jaccard Distance = .972), with most of the dopamine-reward tract’s voxels falling outside of the FMI (D-R Unique Voxels = 95.28%). Qualitatively, the dopamine-reward tract proceeded lateral to the FMI.

**Uncinate Fasciculus (UF).** The dopamine-reward tract exhibited no spatial consistency and complete independence from the UF ($\kappa$ **=** 0.00, Jaccard Distance = 1.00, D-R Unique Voxels = 100%; see Supporting Figure 12). Also as expected, the dopamine-tract remained medial to UF and terminated superior to it.

*Microstructure-Behavior: Localized FA Alterations & MA^+^ Depression Symptoms*

Significant associations were not observed between FA within the ALIC Bundle (*r* = .075, *p =* .477) or mOFC Termination Bundle (*r* = -.149, *p* = .163) and MA^+^ participants’ PHQ9 scores.

**Supporting Figures**

**Supporting Figure 1. Midbrain Seed Creation via Gene-Function Colocalization. (A)** Liberally-thresholded gene-expression and “reward” association metanalysis maps provided by the Neurosynth platform. **(B)** The midbrain presynaptic dopamine seed volume was identified by colocalizing thresholded (*z* $\geq$ 1.5, *k* $\geq$ 50 contiguous voxels) gene-expression and “reward” metanalysis maps (*z* $\geq$ 6, *k* $\geq$ 100 contiguous voxels). Metanalyses in **(A)** and **(B)** overlaid on high-resolution structural image to provide additional anatomical detail. DDC = dopa decarboxylase; VMAT2 = vesicular monoamine transporter (*SLC18A2*); TH = tyrosine hydroxylase.

**Supporting Figure 2. Group-level Tract Reconstruction Probabilities & Final Tract.** *Left & Center:* Probability maps of voxels from tractography protocol for MA^+^ and MA^-^. *Right:* Illustrates the final mesocorticolimbic dopamine-reward tract recovered from applying consistency-thresholding across both MA^+^ and MA^-^ group probability maps. Tract maps and T_1_ reference smoothed for display.

**Supporting Figure 3. Group-level Primary Diffusion Directions within Tract.** Primary diffusion direction vectors averaged across group members within the recovered mesocorticolimbic dopamine-reward tract. *X* = Right-Left (red), *Y* = Anterior-Posterior (green), *Z* = Interior-Superior (blue). (**A)** Axial slices by group. **(B)** Midsagittal-Left lateral slices. **(C)** Midsagittal-Right lateral slices. Diffusion data overlaid on high-resolution T_1_ image to provide additional anatomical detail.

**Supporting Figure 4. Spheroid Parcellations Contained within Tract.** 3D-renderings of the recovered mesocorticolimbic dopamine-reward tract (*red* [50% opacity]) and the 62 discrete spheroids (i.e., along-tract nodes) shaped within its margins. No spheroid fell outside of/extended beyond tract margins. Midbrain presynaptic dopamine seed volume (*yellow*) and reward-sensitive medial orbitofrontal cortex target (*fuchsia*) also illustrated. Renderings dilated and smoothed for display.

**Supporting Figure 5. Anatomical Context of Midbrain Termination Bundle Segment.** *Top Left:* Axial view of Midbrain Termination Bundle bilateral posterior origin. *Top Right:* Coronal view of bilateral posterior origin. *Bottom Left:* Coronal view of bilateral anterior endpoint. *Bottom Right:* Right-sagittal view of bundle long axis. Gray-matter labels from manually-labeled subcortical atlas.^15^ Tract and gray-matter labels smoothed for display and overlayed on high-resolution T_1_ image to provide additional anatomical detail. SNr = substantia nigra pars reticulata; SNc = substantia nigra pars compacta; VTA = ventral tegmental area; PPN = parabrachial pigmented nucleus; GPi = globus pallidus internal segment; GPe = globus pallidus external segment; Hypothal = hypothalamus.

**Supporting Figure 6. Anatomical Context of ALIC Bundle Segment.** *Top Left:* Coronal view of bilateral ALIC Bundle posterior origin. *Top Center & Right:* Left- and right-sagittal views of bundle transition through internal capsule. *Bottom*: Coronal slices of illustrating traversal through internal capsule. Gray-matter labels from manually-labeled subcortical atlas.^15^ Tract and gray-matter labels smoothed for display and overlayed on high-resolution structural image to provide additional anatomical detail. ALIC = anterior limb of internal capsule; GPi = globus pallidus internal segment; GPe = globus pallidus external segment; Hypothal = hypothalamus; NAc = nucleus accumbens

**Supporting Figure 7. Anatomical Context of Extrastriatal Bundle Segment.** *Top:* Axial slices of bilateral Extrastriatal Bundle inferior (*Left*) and superior (*Right*) margins. *Bottom Right:* Rotated 3D-rendering of bundle and proximal structures. *Bottom Center & Left:* Right- and left-sagittal views of bundle long axis. Gray-matter labels from manually-labeled subcortical atlas.^15^ Tract and gray-matter labels smoothed for display and overlayed on high-resolution structural image to provide additional anatomical detail. Renderings dilated and smoothed for display. NAc = nucleus accumbens.

**Supporting Figure 8. Anatomical Context of mOFC Termination Bundle Segment.** *Top:* Axial slices of bilateral anterior margins of mOFC Termination Bundle. *Bottom:* Left- and right-sagittal views showing vertical expansion of anterior aspects of mOFC Termination Bundle intersecting with multiple white-matter parcellation boundaries. Gray-matter labels from manually-labeled subcortical atlas^15^; white-matter parcellations from *Freesurfer’s wmparc* atlas. Tract and gray-matter labels smoothed for display and overlayed on high-resolution structural image to provide additional anatomical detail. mOFC = medial orbitofrontal cortex.

**Supporting Figure 9. Raw Along Tract Distance & Fractional Anisotropy Scatterplot.** Illustrates a shared, higher-order relationship between FA and along tract distance for both groups independently and without the mixed-effects modeling described in the Main Text. Dots reflect individual (unmodeled) FA data points from each of the 62 nodes collected from every participant. Trendlines computed for each group’s data independently via cubic least-squares, with shaded contours reflecting each trend’s 95% confidence interval. Trendline fit was significant for both groups. MA^+^: $R_{adj}^{2}$= .236, *p* < .001; MA^-^: $R_{adj}^{2}$= .197, *p* < .001. FA = fractional anisotropy.

**Supporting Figure 10. Specificity Comparison: Anterior Thalamic Radiations. (A)** Axial slices of recovered mesocorticolimbic dopamine-reward tract spanning inferior to superior bilateral endpoints overlayed with the recovered anterior thalamic radiation tract and their intersection map. **(B)** *Left:* Rotated 3D-renderings of tracts and their intersection points (dilated and smoothed for display) *Right:* Coronal slices illustrating tracts exiting (anterior), traversing, and entering (posterior) anterior limb of internal capsule.

**Supporting Figure 11. Specificity Comparison: Forceps Minor.** Axial slices of the recovered mesocorticolimbic dopamine-reward tract spanning inferior to superior bilateral endpoints overlayed with the recovered forceps minor tract and their intersection map.

**Supporting Figure 12. Specificity Comparison: Uncinate Fasciculus.** Axial slices of the recovered mesocorticolimbic dopamine-reward tract spanning inferior to superior bilateral endpoints overlayed with the recovered forceps minor tract. No voxels intersected between these tracts.

*Supporting Table 1. Detailed MA^+^ Group Substance Use Characteristics*

Urine Drug Screen (% positive)

Methamphetamine	78.26

THC	58.70

Non-meth. or non-THC Combined	15.22

30d Substance Use or Misuse (% endorsing)

Methamphetamine	86.98

Cannabis	67.39

Cocaine	14.13

Hallucinogens	17.39

Rx Opioids	17.39

Rx Sedatives	16.30

Rx Stimulants	11.96

Non-meth. or non-cannabis Combined	45.65

30d Frequency/Day (Mean/SEM)

Methamphetamine	02.89	(0.43)

Cannabis	01.51	(0.32)

Lifetime Substance Use or Misuse (% endorsing)

Methamphetamine	100.0

Cannabis	98.91

Cocaine	89.13

Hallucinogens	82.61

Rx Opioids	52.17

Rx Sedatives	42.39

Rx Stimulants	43.48

Inhalants	32.61

Street Opioids	32.61

**Supporting References**

1.	Yarkoni T, Poldrack RA, Nichols TE, Van Essen DC, Wager TD. Large-scale automated synthesis of human functional neuroimaging data. *Nat Methods*. 2011;8(8):665-670. doi:10.1038/nmeth.1635

2.	Tisdall MD, Hess AT, Reuter M, Meintjes EM, Fischl B, Van Der Kouwe AJW. Volumetric navigators for prospective motion correction and selective reacquisition in neuroanatomical MRI. *Magn Reson Med*. 2012;68(2):389-399. doi:10.1002/mrm.23228

3.	Esteban O, Markiewicz CJ, Blair RW, et al. fMRIPrep: a robust preprocessing pipeline for functional MRI. *Nat Methods*. 2019;16(1):111-116. doi:10.1038/s41592-018-0235-4

4.	Cox RW. AFNI: Software for Analysis and Visualization of Functional Magnetic Resonance Neuroimages. *Comput Biomed Res*. 1996;29(3):162-173. doi:10.1006/cbmr.1996.0014

5.	Cordero-Grande L, Christiaens D, Hutter J, Price AN, Hajnal JV. Complex diffusion-weighted image estimation via matrix recovery under general noise models. *NeuroImage*. 2019;200:391-404. doi:10.1016/j.neuroimage.2019.06.039

6.	Andersson JLR, Skare S, Ashburner J. How to correct susceptibility distortions in spin-echo echo-planar images: application to diffusion tensor imaging. *NeuroImage*. 2003;20(2):870-888. doi:10.1016/S1053-8119(03)00336-7

7.	Andersson JLR, Graham MS, Zsoldos E, Sotiropoulos SN. Incorporating outlier detection and replacement into a non-parametric framework for movement and distortion correction of diffusion MR images. *NeuroImage*. 2016;141:556-572. doi:10.1016/j.neuroimage.2016.06.058

8.	Andersson JLR, Graham MS, Drobnjak I, Zhang H, Filippini N, Bastiani M. Towards a comprehensive framework for movement and distortion correction of diffusion MR images: Within volume movement. *NeuroImage*. 2017;152:450-466. doi:10.1016/j.neuroimage.2017.02.085

9.	Andersson JLR, Graham MS, Drobnjak I, Zhang H, Campbell J. Susceptibility-induced distortion that varies due to motion: Correction in diffusion MR without acquiring additional data. *NeuroImage*. 2018;171:277-295. doi:10.1016/j.neuroimage.2017.12.040

10.	Andersson JLR, Sotiropoulos SN. An integrated approach to correction for off-resonance effects and subject movement in diffusion MR imaging. *NeuroImage*. 2016;125:1063-1078. doi:10.1016/j.neuroimage.2015.10.019

11.	Bastiani M, Cottaar M, Fitzgibbon SP, et al. Automated quality control for within and between studies diffusion MRI data using a non-parametric framework for movement and distortion correction. *NeuroImage*. 2019;184:801-812. doi:10.1016/j.neuroimage.2018.09.073

12.	Hernández M, Guerrero GD, Cecilia JM, et al. Accelerating Fibre Orientation Estimation from Diffusion Weighted Magnetic Resonance Imaging Using GPUs. Yacoub E, ed. *PLoS ONE*. 2013;8(4):e61892. doi:10.1371/journal.pone.0061892

13.	Hernandez-Fernandez M, Reguly I, Jbabdi S, Giles M, Smith S, Sotiropoulos SN. Using GPUs to accelerate computational diffusion MRI: From microstructure estimation to tractography and connectomes. *NeuroImage*. 2019;188:598-615. doi:10.1016/j.neuroimage.2018.12.015

14.	Woolrich MW, Jbabdi S, Patenaude B, et al. Bayesian analysis of neuroimaging data in FSL. *NeuroImage*. 2009;45(1):S173-S186. doi:10.1016/j.neuroimage.2008.10.055

15.	Pauli WM, Nili AN, Tyszka JM. A high-resolution probabilistic in vivo atlas of human subcortical brain nuclei. *Sci Data*. 2018;5(1):180063. doi:10.1038/sdata.2018.63

16.	Park HR, Kim IH, Kang H, et al. Electrophysiological and imaging evidence of sustained inhibition in limbic and frontal networks following deep brain stimulation for treatment refractory obsessive compulsive disorder. Nakamae T, ed. *PLOS ONE*. 2019;14(7):e0219578. doi:10.1371/journal.pone.0219578

17.	Lebel C, Beaulieu C. Longitudinal Development of Human Brain Wiring Continues from Childhood into Adulthood. *J Neurosci*. 2011;31(30):10937-10947. doi:10.1523/JNEUROSCI.5302-10.2011

18.	Samanez-Larkin GR, Levens SM, Perry LM, Dougherty RF, Knutson B. Frontostriatal White Matter Integrity Mediates Adult Age Differences in Probabilistic Reward Learning: Figure 1. *J Neurosci*. 2012;32(15):5333-5337. doi:10.1523/JNEUROSCI.5756-11.2012

19.	Wanigasekera V, Lee MC, Rogers R, et al. Baseline reward circuitry activity and trait reward responsiveness predict expression of opioid analgesia in healthy subjects. *Proc Natl Acad Sci*. 2012;109(43):17705-17710. doi:10.1073/pnas.1120201109

20.	Smillie LD, Jackson CJ, Dalgleish LI. Conceptual distinctions among Carver and White’s (1994) BAS scales: A reward-reactivity versus trait impulsivity perspective. *Personal Individ Differ*. 2006;40(5):1039-1050. doi:10.1016/j.paid.2005.10.012

21.	Chat IKY, Dunning EE, Bart CP, et al. The Interplay Between Reward-Relevant Life Events and Trait Reward Sensitivity in Neural Responses to Reward Cues. *Clin Psychol Sci*. 2022;10(5):869-884. doi:10.1177/21677026211056627

22.	Taubitz LE, Pedersen WS, Larson CL. BAS Reward Responsiveness: A unique predictor of positive psychological functioning. *Personal Individ Differ*. 2015;80:107-112. doi:10.1016/j.paid.2015.02.029

23.	Kroenke K, Spitzer RL, Williams JBW. The PHQ-9: Validity of a brief depression severity measure. *J Gen Intern Med*. 2001;16(9):606-613. doi:10.1046/j.1525-1497.2001.016009606.x

24.	Spitzer RL, Kroenke K, Williams JBW, Löwe B. A Brief Measure for Assessing Generalized Anxiety Disorder: The GAD-7. *Arch Intern Med*. 2006;166(10):1092. doi:10.1001/archinte.166.10.1092

25.	Blevins CA, Weathers FW, Davis MT, Witte TK, Domino JL. The Posttraumatic Stress Disorder Checklist for *DSM‐5* (PCL‐5): Development and Initial Psychometric Evaluation. *J Trauma Stress*. 2015;28(6):489-498. doi:10.1002/jts.22059

26.	Hoeppner BB, Stout RL, Jackson KM, Barnett NP. How good is fine-grained Timeline Follow-back data? Comparing 30-day TLFB and repeated 7-day TLFB alcohol consumption reports on the person and daily level. *Addict Behav*. 2010;35(12):1138-1143. doi:10.1016/j.addbeh.2010.08.013

27.	Mahoney JJ, Kalechstein AD, De La Garza R, Newton TF. Presence and Persistence of Psychotic Symptoms in Cocaine‐ versus Methamphetamine‐Dependent Participants. *Am J Addict*. 2008;17(2):83-98. doi:10.1080/10550490701861201

28.	Kapur BM, Aleksa K. What the lab can and cannot do: clinical interpretation of drug testing results. *Crit Rev Clin Lab Sci*. 2020;57(8):548-585. doi:10.1080/10408363.2020.1774493

29.	Napper LE, Fisher DG, Johnson ME, Wood MM. The reliability and validity of drug users’ self reports of amphetamine use among primarily heroin and cocaine users. *Addict Behav*. 2010;35(4):350-354. doi:10.1016/j.addbeh.2009.12.006

30.	Nelson CB, Rehm J, Bedirhan T, Grant B, Chatterji S. Factor structures for DSM‐IV substance disorder criteria endorsed by alcohol, cannabis, cocaine and opiate users: results from the WHO reliability and validity study. *Addiction*. 1999;94(6):843-855. doi:10.1046/j.1360-0443.1999.9468438.x

31.	Warrington S, Bryant KL, Khrapitchev AA, et al. XTRACT - Standardised protocols for automated tractography in the human and macaque brain. *NeuroImage*. 2020;217:116923. doi:10.1016/j.neuroimage.2020.116923

32.	Hubbard NA, Hutchison JL, Turner MP, et al. Asynchrony in executive networks predicts cognitive slowing in multiple sclerosis. *Neuropsychology*. 2016;30(1):75-86. doi:10.1037/neu0000202

33.	Hubbard NA, Turner MP, Ouyang M, et al. Calibrated imaging reveals altered grey matter metabolism related to white matter microstructure and symptom severity in multiple sclerosis. *Hum Brain Mapp*. 2017;38(11):5375-5390. doi:10.1002/hbm.23727

34.	Sretavan K, Braun H, Liu Z, et al. A Reproducible Pipeline for Parcellation of the Anterior Limb of the Internal Capsule. *Biol Psychiatry Cogn Neurosci Neuroimaging*. 2024;9(12):1249-1261. doi:10.1016/j.bpsc.2024.07.008

35.	Skandalakis GP, Komaitis S, Kalyvas A, et al. Dissecting the default mode network: direct structural evidence on the morphology and axonal connectivity of the fifth component of the cingulum bundle. *J Neurosurg*. 2021;134(4):1334-1345. doi:10.3171/2020.2.jns193177

36.	Skandalakis GP, Neudorfer C, Payne CA, et al. Establishing connectivity through microdissections of midbrain stimulation-related neural circuits. *Brain*. 2024;147(9):3083-3098. doi:10.1093/brain/awae173
